# Seasonal variation of ambulatory blood pressure in Chinese hypertensive adolescents

**DOI:** 10.3389/fped.2022.1022865

**Published:** 2022-11-18

**Authors:** Yi Zhou, Lin Zhao, Xu Meng, Qiu-Jing Cai, Xiao-Lei Zhao, Xian-Liang Zhou, Ai-Hua Hu

**Affiliations:** ^1^ Department of Cardiology, Fuwai Hospital, Chinese Academy of Medical Sciences and Peking Union Medical College, National Center for Cardiovascular Diseases, Beijing, China; ^2^ Department of Non-Communicable Disease Management, Beijing Children's Hospital, Capital Medical University, National Center for Children's Health, Beijing, China

**Keywords:** seasonal variation, ambulatory blood pressure monitor, adolescents, dipping pattern, white coat hypertension

## Abstract

**Background:**

Blood pressure (BP) exhibits seasonal variation with lower levels at higher temperatures and vice versa. This phenomenon affects both sexes and all age groups. So far, only a few research studies have investigated this condition in adolescents and none of them were based on hypertensive population or ever applied ambulatory blood pressure monitor (ABPM). Therefore, we carried out the first study that used ABPM to record seasonal variation of blood pressure in hypertensive adolescents.

**Methods:**

From March 2018 to February 2019, 649 ABPMs from hypertensive adolescents between 13 and 17 years who were referred to wear an ABPM device in Beijing and Baoding were extracted. Seasonal change in ambulatory BP value, dipping status, and prevalence of different BP phenotypes were analyzed and compared.

**Results:**

Mean age of participants was 14.9 ± 1.5 years and 65.8% of them were boys. Of the participants, 75.3% met the criteria of overweight or obesity. From summer to winter, average 24-hour, day-time, and night-time BP showed significant rise, which was 9.8/2.8, 9.8/3.0, and 10.9/3.4 mmHg, respectively. This seasonal effect on BP was not dependent on the obesity degree. In addition, higher prevalence of nondippers and risers existed in winter while white coat hypertension was more frequent in warmer seasons.

**Conclusion:**

Hypertensive adolescents showed evident seasonal change in their ABPM results, which was featured by elevated BP level and more frequent abnormal dipping patterns in winter. On the contrary, higher prevalence of white coat hypertension was found in warmer seasons. Physicians should take seasonal variation into consideration when managing adolescent hypertension.

## Introduction

Seasonal variation in blood pressure (BP) is a global phenomenon. Compared with summer, winter usually witnesses higher prevalence and greater severity of hypertension (1, 2). This seasonal variation in blood pressure affects both men and women, young and old, normotensive individuals and hypertensive patients, and contributes to the increased cardiovascular risk in winter (3). In recent years, consensus recommendation and updated guideline have been provided for physicians to handle this situation in adult hypertensive patients (4, 5).

However, when referring to children and adolescent hypertension, similar documents are yet to be available due to insufficient data. To date, only a handful of research studies in western countries and India have investigated seasonal BP variation in youth and majority of their participants were normotensive subjects (6–10). Therefore, these results may not be completely transferable to hypertensive adolescents. In addition, none of the researchers above have ever used ambulatory blood pressure monitor (ABPMs) in their studies. Compared with clinic measurement, ABPMs appeared to be better in reflecting blood pressure level and predicting target organ damage (TOD) (11, 12). In addition, it provided necessary information allowing the physician to evaluate nocturnal dipping status and identify specific BP phenotypes such as white coat hypertension (WCH) and masked hypertension (MHT), which, according to adult studies, were also affected by seasonality (13, 14).

The goal of the present study was to perform a more comprehensive assessment of seasonal BP variation among hypertensive adolescents. Therefore, ABPM was applied in our study, which could not only record the ambulatory BP level but also reveal dipping status and classify BP phenotype.

## Materials and methods

### Study design and population

Present study was carried out in Beijing and Baoding, a city in Hebei Province. From March 2018 to February 2019, adolescents aged 13–17 years who were referred to wear an ABPM device produced by Kang Electronics Technology Company were recruited. Exclusion criteria were as follows: (1) adolescents with normal clinic BP value (<130/80 mmHg) and normal ambulatory BP value (24-h < 125/75 mm Hg, day-time <130/80 mm Hg, and night-time <110/65 mm Hg) (15); (2) with known history of antihypertensive drugs; (3) with known history of diseases that could affect BP (chronic kidney disease, congenital heart disease, thyroid disorder, Cushing's syndrome, etc.). Finally, 649 hypertensive adolescents were recruited and their first-time ABPMs during the studied period were extracted. Detailed information about the study was given to the participants and his/her family and informed consent was obtained from the family. This study was conducted in accordance with the Declaration of Helsinki and was approved by the Ethics Committee of Beijing Hypertensive League.

### Office and ambulatory BP measurement

Office BP was measured in a sitting position on the right arm by trained medical attendant using an automatic electronic oscillometric sphygmomanometer (OMRON HBP-1300, Kyoto, Japan). It was measured three times with a 5-minute interval and the mean value was recorded. Heart rate (HR) was tested simultaneously using the same device. Ambulatory BP was monitored by a portable, noninvasive, automated monitoring and recording system (KC-2300A; Beijing Kangkang Shengshi Information Technology Co, Ltd, China). It was programmed to record systolic blood pressure (SBP), diastolic blood pressure (DBP), and HR at a 30-minute interval over day time (from 06:00 to 22:00) and at a 60-minute interval during night time (from 22:00 to 06:00). Following clinical practice guideline, the nondominant arm was used for ABPM (16). Qualified ABPMs required at least 70% of successful readings during monitoring period and should include a minimum of one reading per hour. Otherwise, the result would be considered as inappropriate for analysis (15).

The dipping pattern of BP was classified by the percentage decline in night-time SBP compared to day-time SBP. Extra-dipper was defined as nocturnal SBP declined more than 20%, dipper as 20%–10%, nondipper as less than 10%, and riser as elevated night-time SBP.

We categorized BP phenotypes based on an updated statement on pediatric ABPM from the American Heart Association (AHA), which simplified the classification of BP phenotypes among subjects ≥13 years by using static cut points instead of percentile (15). Definition of normal blood pressure has been described in the exclusion criteria. MHT was defined as elevated ambulatory BP (24 h BP ≥ 125/75 mmHg, day-time BP ≥ 130/80 mmHg, or night-time BP ≥ 110/65 mmHg) despite normal clinic BP (<130/80 mmHg). WCH was defined as elevated clinic BP (≥130/80 mmHg) and nonelevated ambulatory BP (24 h BP < 125/75 mmHg, day-time BP < 130/80 mmHg, and night-time BP < 110/65 mmHg). Sustained hypertension (SHT) was defined as both elevated clinic and ambulatory BP.

### Other assessments and seasonal factors

Basic demographic information including age, gender, height, and weight was extracted from the database of ABPM reports. Body mass index (BMI) was calculated as weight (kg) divided by height squared (m^2^). Adolescents were further grouped into categories based on BMI: normal (<85th percentile), overweight (≥85th and <95th percentile), and obesity (≥95th percentile) (17).

**Figure 1:**
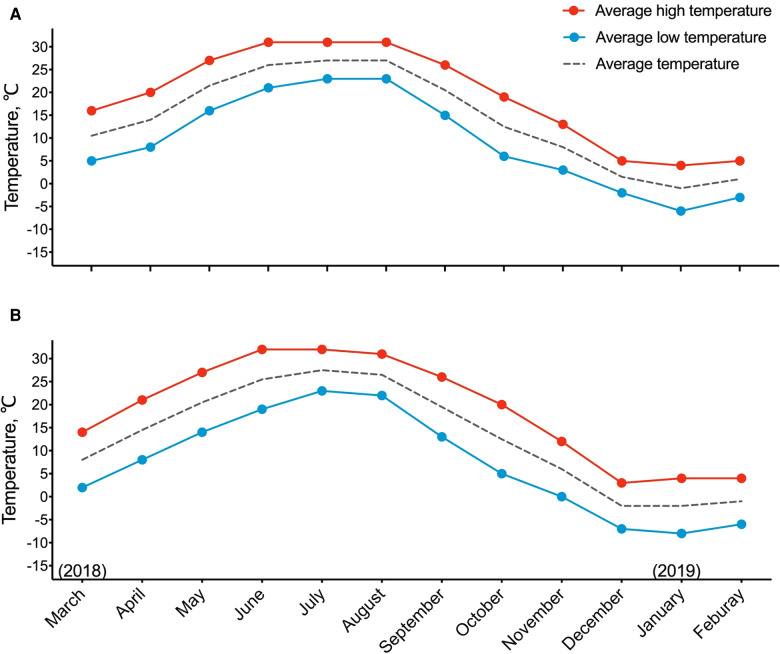
Temperature trend across the seasons during studied period in Beijing (**A**) and Baoding (**B**). Season was defined as follows: summer included June, July, and August; winter included December, January, and February; middle season included March, April, May, September, October, and November.

Beijing and Baoding were two adjacent cities located in northeastern China. Affected by temperature and continental monsoon climate, both cities had four distinct seasons. Monthly average temperature during our study period was presented in Figure 1. Each season was defined as follows: summer ranged from 1 June to 31 August; winter ranged from 1 December to the last day of following year's February; spring ranged from 1 March to 31 May, and autumn ranged from 1 September to 31 November. Since most studies on seasonal BP variation focused on the hottest and the coldest season, spring and autumn were merged into one group named middle season in the present study. The average temperature of different seasons in Beijing/Baoding was 26.7°C/26.5°C in summer, 14.5°C/16.5°C in middle season, and 0.5°C/−1.6°C in winter.

### Statistical analysis

For all characteristics, continuous variables were expressed as mean ± SD and categorical variables were expressed as number (%). Statistical significance was assessed using the Chi-squared test (for categorical variables) and ANOVA method (for continuous variables with homogenous variance) or nonparametric test (for continuous variables with heterogeneous variance). Multivariable linear regression models were used to assess the independent effect of seasonal factors on ambulatory SBP level, which were adjusted for host factors (age, gender, BMI) and office SBP. All statistical analyses were performed using SPSS, version 26.0 (SPSS, Inc., Chicago, IL, United States), and a two-sided *P *< 0.05 was considered statistically significant.

## Results

### Clinical characteristic of participants

Table 1 showed participants’ basic characteristics. Among 649 adolescents, the mean age was 14.9 ± 1.5 years and 65.8% were boy. 277 ABPMs were recorded in middle season while 190 were in summer and 182 were in winter. Compared with other seasons, BMI level was higher among adolescents participated in winter (summer: 25.8 ± 4.9 kg/m^2^, middle: 26.2 ± 5.0 kg/m^2^, winter: 27.6 ± 5.6 kg/m^2^, *P *< 0.001). Distribution of ABPMs in different months during studied period is presented in Supplementary Figure S1.

**Table 1:** Demographic characteristics and BP findings of participants in each season.

	Summer (n = 190)	Middle (n = 277)	Winter (n = 182)	*P*-value
Age, years	15.4 ± 1.3^a^^c^	14.6 ± 1.4^a^^b^	14.9 ± 1.5^b^^c^	<0.001
Male, %	63.7	65.3	68.7	0.584
BMI, kg/m^2^	25.8 ± 4.9^a^	26.2 ± 5.0^a^	27.6 ± 5.6^b^^c^	<0.001
Office SBP, mmHg	141.0 ± 14.5^a^	142.5 ± 12.4^a^	151.5 ± 15.2^b^^c^	<0.001
Office DBP, mmHg	90.04 ± 9.8	90.3 ± 11.0	88.4 ± 13.3	0.254
24 h SBP, mmHg	125.4 ± 9.3^a^	125.6 ± 8.5^a^	135.2 ± 10.3^b^^c^	<0.001
24 h DBP, mmHg	75.0 ± 8.0^a^	74.4 ± 7.3^a^	77.8 ± 8.7^b^^c^	<0.001
24 h HR, beats/min	77.2 ± 8.0	76.0 ± 8.5	76.1 ± 8.6	0.327
Day-time SBP, mmHg	127.4 ± 9.6^a^	128.0 ± 8.9^a^	137.2 ± 10.4^b^^c^	<0.001
Day-time DBP, mmHg	77.1 ± 8.2^a^	76.8 ± 7.5^a^	80.1 ± 8.9^b^^c^	<0.001
Day-time HR, beats/min	80.3 ± 8.7	79.2 ± 8.9	79.5 ± 9.1	0.464
Night-time SBP, mmHg	116.6 ± 10.5^a^	116.6 ± 10.6^a^	127.5 ± 13.0^b^^c^	<0.001
Night-time DBP, mmHg	65.6 ± 9.4^a^	64.9 ± 9.1^a^	69.0 ± 10.0^b^^c^	<0.001
Night-time HR, beats/min	63.1 ± 9.1^a^	63.1 ± 10.3^a^	65.2 ± 9.2^b^^c^	0.043

BMI, body mass index; SBP, systolic blood pressure; DBP, diastolic blood pressure; HR, heart rate

Statistical significance was defined as P < 0.05.

^a^ Statistical significant in seasonal differences vs. winter.

^b^ Statistical significant in seasonal differences vs. summer.

^c^ Statistical significant in seasonal differences vs. middle.

### Seasonal variation in office and ambulatory BP value

**Figure 2:**
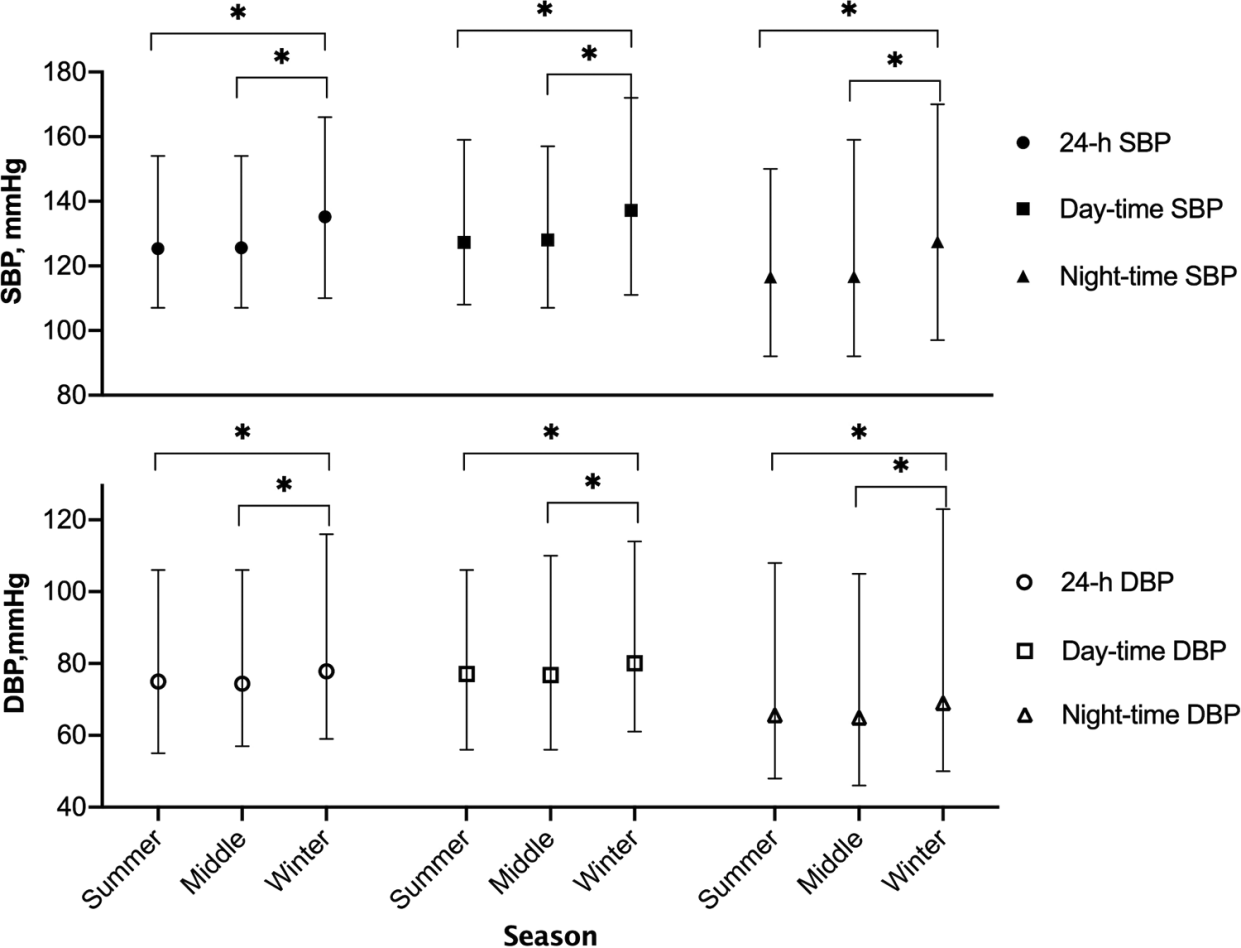
Seasonal variation of 24-hour, day-time, and night-time blood pressure. Black and white points in this figure indicated SBP and DBP, respectively. SBP, systolic blood pressure; DBP, diastolic blood pressure. *P < 0.05.

Office SBP was higher in winter than in other seasons while office DBP exhibited no statistical difference. Ambulatory monitor showed both day-time and night-time BP were significantly higher in winter and the difference was larger in SBP than in DBP. Clinic and ambulatory BP values were also shown in Table 1, and the 24-hour, day-time, and night-time SBP and DBP parameters in each season were shown in Figure 2.

**Figure 3:**
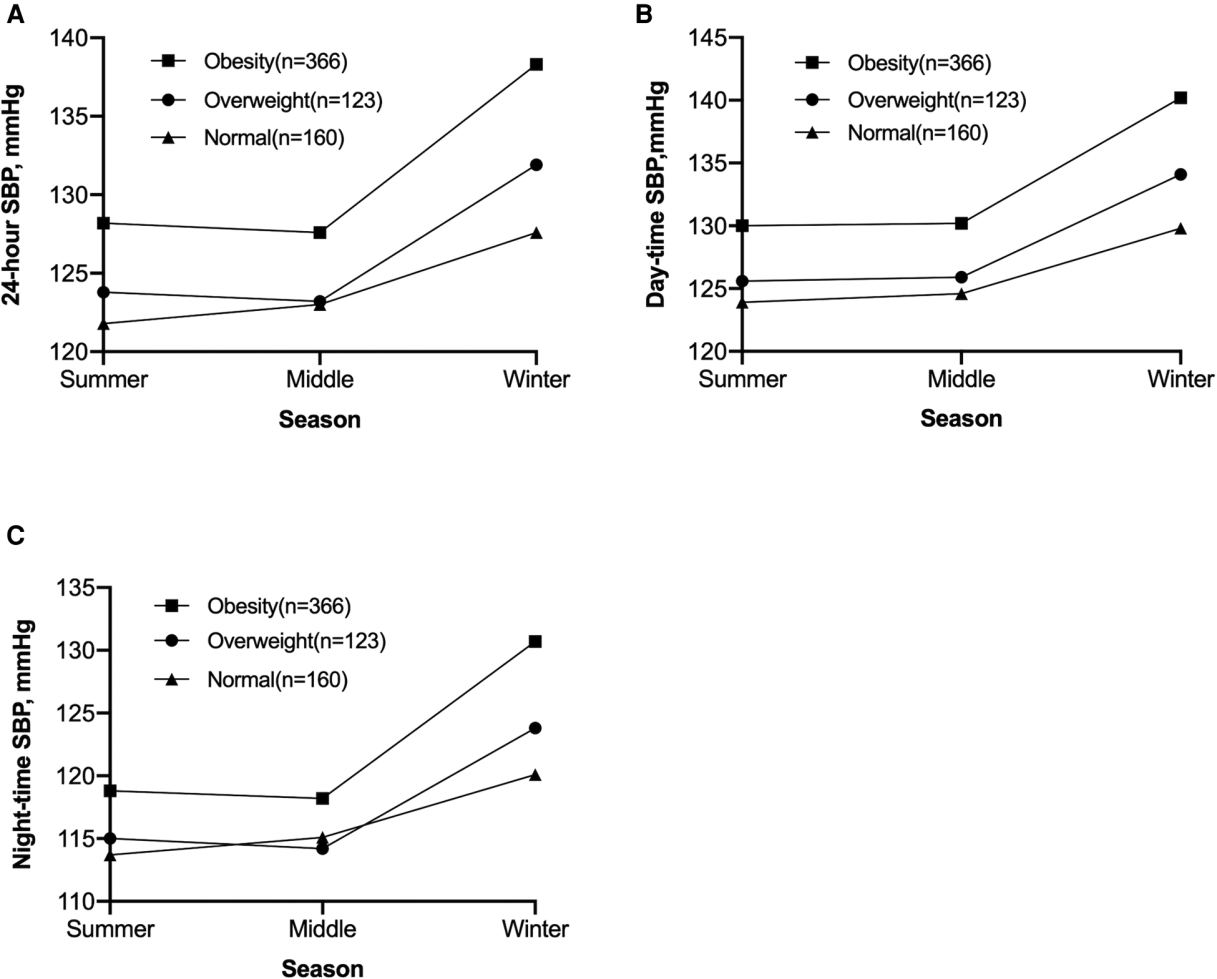
Seasonal variation of 24-hour SBP (**A**), day-time SBP (**B**), and night-time SBP (**C**) of adolescents with different obesity degree. Result of two-way ANOVA showed no significant interaction effect between obesity degree and seasonal variation in 24-hour SBP (*P *= 0.085), day-time SBP (*P *= 0.270), or night-time SBP (*P *= 0.084). SBP, systolic blood pressure.

For overweight (*n* = 123) and obese (*n* = 366) subjects, a secondary analysis was performed to examine the relationship between severity of obesity and seasonal variation in BP level. The result was shown in Figure 3. Ambulatory SBP was obviously higher during winter in all three groups, and this change seemed to be more apparent among overweight and obese subjects. Nevertheless, two-way ANOVA showed no significant interaction between obesity degree and seasonal effect on 24-hour SBP (*P *= 0.085), day-time SBP (*P *= 0.270), or night-time SBP (*P *= 0.084).

 Table 2 showed the result of multivariable linear regression analysis between seasonal factors (winter vs. others) and ambulatory BP value. After adjusting for host factors (age, gender, and BMI) and office SBP, the coefficient of winter remained significantly positive in 24-hour, day-time, and night-time SBP.

**Table 2:** Multivariable linear regression analysis for 24-hour, day-time, and night-time SBP.

	24-hour SBP			Day-time SBP			Night-time SBP		
	β	B (95% CI)	*P*	β	B (95% CI)	*P*	Β	B (95% CI)	*P*
Winter (vs. other seasons)	0.248	5.663 (4.374–6.952)	<0.001	0.226	5.231 (3.931–6.531)	<0.001	0.292	7.994 (6.162–9.872)	<0.001
Age	0.042	0.294 (-0.091–0.679)	0.134	0.198	0.074 (-0.314–0.463)	0.707	0.044	0.375 (-0.173–0.922)	0.180
Male	0.167	3.606 (2.440–4.772)	<0.001	0.159	3.493 (2.317–4.668)	<0.001	0.157	4.826 (2.417–5.732)	<0.001
BMI	0.298	0.594 (0.482–0.679)	<0.001	0.285	0.575 (0.463–0.688)	<0.001	0.264	0.630 (0.472–0.788)	<0.001
Office SBP	0.424	0.300 (0.259–0.341)	<0.001	0.462	0.332 (0.290–0.373)	<0.001	0.213	0.181 (0.123–0.239)	<0.001

BMI, body mass index, SBP, systolic blood pressure.

Table 2 shows the multivariable linear regression analysis of seasonal factors (winter vs. others) for 24-hour, day-time, and night-time home SBP. The following adjusted factors were included: age, sex, body mass index, and office SBP level. β and B indicate the standardized coefficient and nonstandardized coefficient, respectively.

### Dipping status of BP in each season

**Figure 4:**
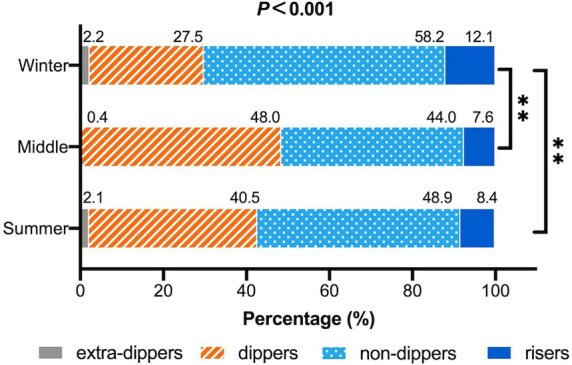
Seasonal variation in dipping patterns of blood pressure. Result of chi-square test was listed above the columns and result of multiple comparisons was listed on the right side. Bonferroni correction was performed in multiple comparison. *P < 0.05; **P < 0.01.

Nocturnal dipping status of participants in each season was presented in Figure 4. Abnormal circadian patterns including nondipper and riser were more popular in winter (summer 57.4%; middle 51.6%; winter 70.3%; *P *< 0.001), whereas dipper appeared to be more common in summer and middle seasons (summer 40.5%; middle 48.0%; winter 27.5%; *P *< 0.001). In multivariate logistic regression analysis, there was similar association between seasonal factors (winter vs. others) and abnormal circadian pattern after adjusted for age, gender, and BMI (OR: 1.982, 95% CI: 1.369–2.871, *P *< 0.001) (Supplementary Table S1). As for extra-dipper, the number was too small to prove any statistical difference.

### Seasonal variation in the prevalence of different BP phenotypes

**Figure 5:**
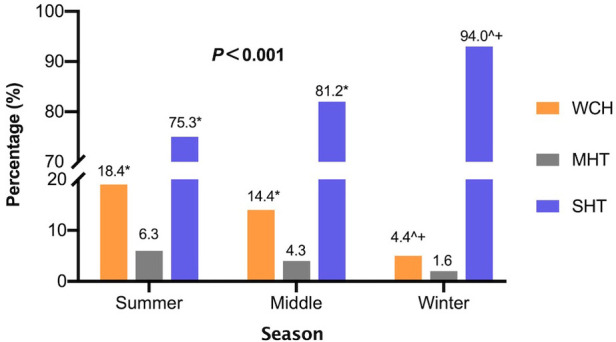
Prevalence of different BP phenotypes in each season. Chi-square test and Bonferroni correction for multiple comparisons were used. Results of the chi-square test were listed on top and the result of multiple comparison was shown in the following terms: *P < 0.05 vs winter; ^*P *< 0.05 vs. summer; +*P *< 0.05 vs. middle. WCH, white coat hypertension; MHT, masked hyper-tension; SHT, sustained hypertension.

Similar to adults, children and adolescents’ BP status were categorized into four phenotypes: normo-tension, WCH, MHT, and SHT (15, 18). Prevalence of different BP phenotypes in each season is listed in Figure 5. Among 649 hypertensive participants, sustained hypertension prevailed all year round and became more dominant in cold days (winter 94.0%; middle 81.2%; summer 75.3%; *P *< 0.001). On the contrary, the prevalence of WCH was higher in other seasons than in winter (winter 4.4%; middle 14.4%; summer 18.4%; *P *< 0.001). Masked hypertension occupied the least proportion and did not show significant seasonal difference in proportion (winter 1.6%; middle 4.3%; summer 6.3%; *P *= 0.078).

## Discussion

In present study, we used ABPM to investigate the seasonal effect on adolescent hypertension. By applying this measurement, seasonal variation in BP level, nocturnal dipping status, and frequency of BP phenotypes among this particular population were revealed. A summary of our results was as follows: (1) 24-hour, day-time, and night-time BP were higher in winter than other seasons, and this seasonal effect was not dependent on obesity degree. (2) Abnormal dipping patterns were more frequent in winter than in other seasons. (3) BP phenotypes were also affected by seasonality, featured by higher prevalence of white coat hypertension in warmer seasons.

In present study, average ambulatory SBP increased by 9.8 mmHg from summer to winter and DBP increased by 2.8 mmHg. A similar trend has been reported in previous studies but with smaller difference (6, 10). A study of 6,741 children and adolescents in Germany recorded 4.5/2.4 mmHg difference in clinic BP between winter and summer (6). In another study from Turkey, this gap was 5.9/3.6 mmHg (10). Compared with Beijing and Baoding, Germany had a smaller temperature variation between winter and summer, which was less than 20°C (6). Affected by Mediterranean climate, Turkey was also featured by a relatively mild temperature change throughout the year (10). Therefore, smaller seasonal BP difference reported in these areas was reasonable. Previous research also suggested that old age and usage of diuretic might enhance seasonal change in BP (19). These two factors were not investigated in present study due to study design. However, we found that the degree of obesity seemed to have no significant impact on seasonal variation in the BP level.

Several factors contributed to the elevated BP in winter. Sympathetic nerve system responded directly to cold exposure and increased the excretion of plasma norepinephrine level, which caused peripheral blood vessel constriction (20, 21). This physiological change was found in both adults and children (22). In addition, winter was also associated with more energy intake and less activity, which could indirectly cause elevated BP by gaining more weight and fat accumulation in the abdominal region (23, 24). In present study, BMI level was higher among winter participants and proved to be positively related to the ambulatory BP level in multivariable linear regression analysis. Although we did not collect information about subjects’ diet habits, it was reported that cold exposure could enhance salt intake (25). Meanwhile, people tended to sweat less in winter. Considering the role of sodium’s level in BP regulation and the greater salt sensitivity of Asian hypertensive patients, we believed that such seasonal change could also increase BP level in winter (26, 27).

However, elevated BP did not just happen in winter. In a large sample study, Fedecostante et al. found that night-time SBP was slightly higher in summer than winter (28). Later, Narita et al. compared evening BP (measured before going to bed) and night-time BP (measured during sleep) in different seasons and found the summer rise in blood pressure only happened during sleep (14, 29). Therefore, he suggested this phenomenon was probably caused by the hot-induced poor sleep quality in summer. Apart from temperature, Modesti et al. found that seasonal change in physical activity was also likely to affect the variation in night-time BP (30). In present study, night-time BP did not show the expected rise in summer. On the contrary, it changed the same way as day-time BP, getting higher in winter and lower in summer. Even after adjusting for age, gender, and BMI level, winter season remained positively correlated with night-time SBP. Several potential reasons might explain our results. On one hand, researchers admitted that the elevated night-time BP observed in some areas, for example in Italy, could be partly due to the uncommon installation of domestic air conditioners (28). In contrast, air conditioners were much more popular in Beijing and Baoding thus more comfortable sleep and more stable night-time BP in summer could be guaranteed (31). On the other hand, most winter participants in present study received their ABPM in late January so the higher night-time BP might be associated with increasing workload and shorter sleep duration for final exam. This hypothesis was indirectly supported by an elevated heart rate from these adolescents recorded in the same period.

Dipping status of BP was also affected by seasonality. According to previous studies, nondippers and risers were more common in summer because of the decreased day-time BP and the elevated night-time BP in this season (13, 28, 29). Nevertheless, our study showed exactly the opposite way, with a higher percentage of abnormal circadian patterns in winter. Such conflicting results could be considered a further confirmation of the different seasonal change in night-time BP, which has been discussed above. In addition, it was a little surprise to find no statistical relationship between BMI and abnormal dipping status. This could have resulted from selection bias since all subjects in present study were hypertensive and three-quarters of them had weight problems.

In present study, prevalence of white coat hypertension changed in parallel with temperature, which was higher in summer and lower in winter. A similar phenomenon was seen in another study with hypertensive elders from Japan, which also found WCH more frequent in summer (14). Unlike sustained and masked hypertension, whether WCH was associated with target organs damage in children remained controversial (32–34). However, longitudinal data suggested that children with elevated clinic BP were more likely to develop sustained hypertension and this progression could take months to years (32, 35). Therefore, youth with WCH were recommended to take repeated ABPM every 1–2 years (16). Our results suggested that physicians should pay more attention to identify WCH in warmer seasons so that they could arrange proper follow-up visits and avoid unnecessary medication for young patients.

## Limitation

Strengths of this study included the relative appreciable samples and the application of ABPM. In addition, focusing on hypertensive individuals, our result might be better suited to the rising number of adolescents troubled by abnormal BP. Still, there were some limitations. The most obvious one was we did not perform ABPM for the same subject throughout the four seasons given its cross-sectional design. It should also be noticed that most participants in the middle group were recruited in late April and May. Therefore, the result that there was no significant difference between middle season and summer might be partly due to this seasonal bias. Moreover, direct information on seasonal variation in diet, physical activity, and daily schedule was lacking. Questionnaires including these details should be distributed to participants in a future study on the similar topic.

Apart from the cut points we used in the current study, sex/age-specific 95th percentile could also help identify adolescent hypertension. The most widely accepted reference was based on the result from a German study (36). Recently, a database including ABPMs from 1,445 Chinese children aged between 8 and 17 years became available (37). In addition to its convenience, the updated cut points showed comparable performance with 95th percentile in predicting hypertension-related TOD (15). Therefore, we used it to define hypertension in the present study.

## Conclusions

For hypertensive adolescents, seasonal variation could lead to evident change in their ABPM results. In winter, elevated BP value and higher prevalence of abnormal circadian pattern existed, while in summer, things changed toward the opposite way and white coat hypertension became more popular. Most of the seasonal variation observed was in line with literature, but the change in night-time BP and dipping status appeared to be different, which needed further investigation to confirm. Additional studies were also encouraged for guidelines on managing such variation when managing pediatric hypertension.

## Data Availability

The original contributions presented in the study are included in the article/**Supplementary Material**, further inquiries can be directed to the corresponding authors.
