# Supplementary material for: Seasonal variation of ambulatory blood pressure in Chinese hypertensive adolescents
Source: Front Pediatr. 2022 Nov 18;10:1022865. doi: 10.3389/fped.2022.1022865 (PMC9715761; doi:10.3389/fped.2022.1022865)
Supplement: Supplementary file 1 [file Table1.doc]

Supplementary Table1. Multivariate logistic regression analysis for abnormal circadian patterns

|  | Abnormal circadian patterns | |
| --- | --- | --- |
|  | OR (95% CI) | *P* |
| Winter  (vs. other seasons) | 1.982  (1.369-2.871) | ＜0.001 |
| Age | 0.974  (0.873-1.086) | 0.633 |
| Male | 1.257  (0.901-1.752) | 0.178 |
| BMI | 1.009  (0.978-1.042) | 0.563 |

*S Table 1 showed the binary logistic regression analysis between seasonal factors(winter vs. others) and prevalence of abnormal circadian patterns. The following adjusted factors were included: age, sex and body mass index. Abnormal circadian patterns included riser and non-dipper. BMI: body mass index*
