# Supplementary figures and images for: Seasonal variation of ambulatory blood pressure in Chinese hypertensive adolescents
Source: Front Pediatr. 2022 Nov 18;10:1022865. doi: 10.3389/fped.2022.1022865 (PMC9715761; doi:10.3389/fped.2022.1022865)

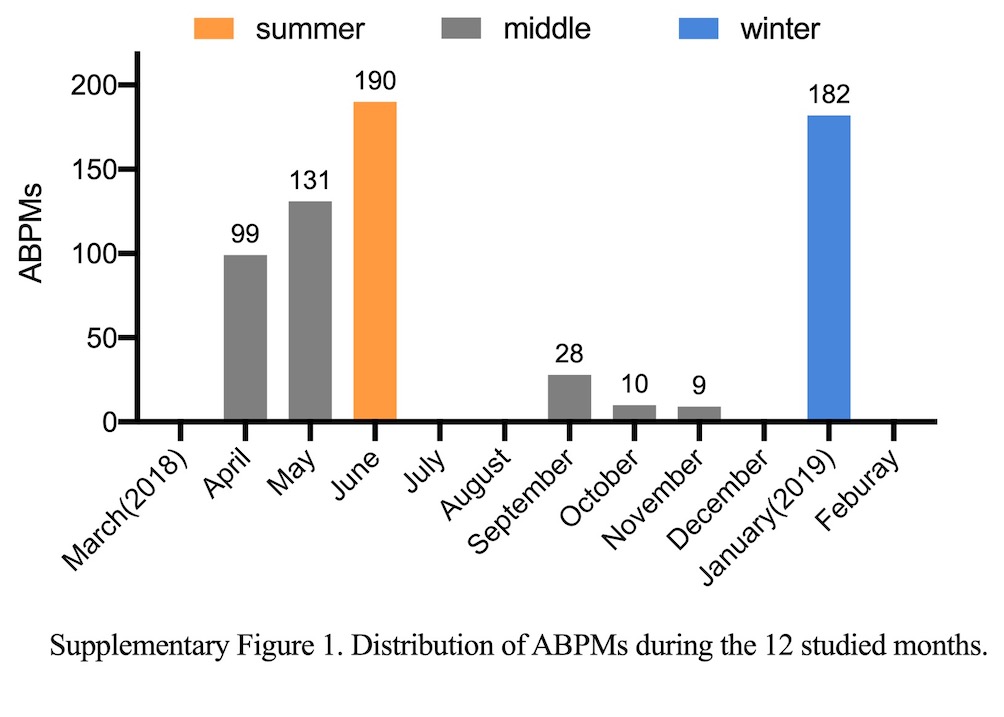

Supplement: Supplementary file 2 [file Image1.jpeg]
